# Advanced Biological Therapies: Principles, Mechanisms and Medical Applications

**DOI:** 10.3390/jcm15176523

**Published:** 2026-08-23

**Authors:** Rafal Brozek, Antonina Lorenz, Barbara Dorocka-Bobkowska, Maciej Kurpisz

**Affiliations:** 1 Department of Prosthodontics and Geriatric Dentistry, Poznan University of Medical Sciences, 60-812 Poznan, Poland; broz@ump.edu.pl (R.B.); bdorocka@ump.edu.pl (B.D.-B.); 2 Department of Reproductive Biology and Stem Cells, Institute of Human Genetics, Polish Academy of Sciences, 60-479 Poznan, Poland; antonina.lorenz@igcz.poznan.pl

**Keywords:** Biopharmaceutics, signaling pathways, cytokines, JAK inhibitors, STAT inhibitors, rheumatic diseases, periodontitis

## Abstract

Biotherapeutics are medicinal agents derived from or related to naturally occurring molecules in the body and are designed to modulate specific immune targets. This review examines clinically established biologics and targeted small molecules that affect cytokine-driven transcriptional programs, principally JAK/STAT and canonical and non-canonical NF-κB signaling, across autoimmune, neoplastic, hematological, dermatological, and rheumatic diseases. Periodontitis is used as a translational model because dysbiotic mucosal inflammation, cytokine signaling, and osteoclastogenesis may also intersect with systemic autoimmunity. In particular, *Porphyromonas gingivalis*-associated protein citrullination provides a plausible link to anti-citrullinated protein antibody-positive rheumatoid arthritis in genetically susceptible individuals, although causality remains unproven. The review also evaluates plant-derived modulators of JAK/STAT and related transcriptional pathways. Curcumin and resveratrol have entered small rheumatoid arthritis studies, whereas evidence for catechins, celastrol, and artemisinin derivatives in autoimmune disease remains predominantly preclinical. These compounds may inform adjunctive or locally delivered strategies, but clinical translation requires better target selectivity, bioavailability, dose standardization, and safety data.

## 1. Introduction

Biological medicines include vaccines, blood-derived products, recombinant proteins, allergens, gene therapies, tissues, and cell-based products obtained from human, animal, or microbial sources or produced by biotechnology. Compared with chemically synthesized small molecules, they are generally more structurally complex and may require specific manufacturing and handling controls [1]. Therapeutic biologics can selectively modulate immune targets, but this specificity does not eliminate the risk of infections or other immune-mediated adverse events [2].

This review focuses on biological therapies and related targeted agents that modulate cytokine-driven JAK/STAT and NF-κB transcriptional programs. Periodontitis is used as a translational case study to illustrate how local dysbiotic inflammation interfaces with systemic autoimmunity, while plant-derived small molecules are discussed as exploratory, non-biologic pathway modulators. The advantages, limitations, and evidence gaps of these approaches are critically evaluated.

Advances in the understanding of intracellular signaling pathways and the role of key signaling molecules in cellular function have substantially contributed to the development of targeted biological therapies [3,4]. Increasing attention has been directed toward signaling networks mediated by immune cells, cell-surface receptors responsible for intercellular communication, intracellular cascades activated by metabolic enzymes, and regulatory mechanisms governing gene expression and membrane permeability. Binding of signaling molecules to their cognate receptors on the surface or within target cells initiates a series of intracellular events that regulate numerous biological processes. Detailed investigation of these pathways has revealed the existence of positive and negative feedback mechanisms, alterations in the release of inflammatory mediators, and opportunities for modulating inflammatory responses through selective interference with pro-inflammatory signaling networks [5].

Inflammation plays a central role in the pathogenesis of numerous diseases. Under physiological conditions, inflammatory responses constitute an essential component of innate immunity, providing protection against infection, tissue injury, and pathogen invasion. However, persistent inflammation or dysregulation of inflammatory signaling pathways may contribute to disease progression, deterioration of tissue function, and, in severe cases, mortality [6]. The high prevalence of chronic inflammatory disorders, including asthma, osteoarthritis, and psoriasis, highlights the close association between immune dysregulation and sustained inflammatory activity. Similar mechanisms have been implicated in the pathogenesis of Crohn’s disease and ulcerative colitis.

Steroidal anti-inflammatory drugs and non-steroidal anti-inflammatory drugs (NSAIDs) remain among the most commonly prescribed therapies for inflammatory diseases. Nevertheless, long-term administration of these agents is associated with a substantial risk of adverse effects. Prolonged corticosteroid treatment may result in hypertension, cataracts, glaucoma, hyperglycemia, and osteoporosis. Similarly, chronic NSAID use has been linked to gastrointestinal complications, including gastric ulceration and gastrointestinal bleeding [7,8].

Cytokines are small signaling proteins produced by immune and stromal cells that coordinate cell activation, differentiation, proliferation, and migration. Their pro-inflammatory, anti-inflammatory, and immunoregulatory actions must remain balanced to prevent persistent inflammation and tissue injury. Tumor necrosis factor-alpha (TNF-α) and interleukin-1 beta (IL-1β) promote leukocyte recruitment and amplify inflammatory mediator production mainly through NF-κB and mitogen-activated protein kinase (MAPK) signaling. IL-6 supports the acute-phase response, B-cell activation, and T helper 17 (Th17) differentiation through JAK/STAT3, whereas IL-17 stimulates epithelial and stromal cells to produce chemokines that recruit neutrophils. IL-12 promotes Th1 differentiation and interferon-gamma (IFN-γ) production, while IL-23 sustains Th17 responses. In contrast, IL-2 supports activated T-cell proliferation and regulatory T-cell homeostasis, and IL-10 limits antigen presentation and pro-inflammatory cytokine production [9,10,11,12].

In periodontitis, dysregulated TNF-α, IL-1β, IL-6, and IL-17 signaling increases matrix metalloproteinase expression and disrupts the RANKL/osteoprotegerin balance, thereby promoting connective tissue degradation and osteoclast-mediated alveolar bone resorption. Conversely, IL-10 and regulatory T cells may restrain local inflammation. These distinct cytokine networks, operating principally through JAK/STAT, NF-κB, and MAPK pathways, provide a mechanistic rationale for targeted biological therapies in chronic inflammatory and autoimmune diseases [13,14,15,16].

Periodontitis is a chronic, multifactorial, biofilm-associated inflammatory disease in which microbial dysbiosis and a dysregulated host response cause loss of periodontal attachment and alveolar bone. Its clinical severity is staged according to the extent of tissue destruction and treatment complexity, whereas grading estimates progression and incorporates major risk modifiers. Periodontitis was selected as a clinical example in this review because it provides an accessible mucosal model of persistent inflammation in which JAK/STAT, NF-κB, MAPK, and RANKL/osteoprotegerin networks converge to regulate cytokine production, connective-tissue breakdown, and osteoclastogenesis [16,17].

The periodontitis-rheumatoid arthritis (RA) relationship is biologically plausible but should not be interpreted as proven causality. *P. gingivalis* expresses peptidylarginine deiminase (PPAD) and arginine gingipains that can generate citrullinated bacterial and host peptides, including fibrinogen and α-enolase. These neoepitopes may promote loss of tolerance and anti-citrullinated protein antibody (ACPA) formation, particularly in individuals carrying HLA-DRB1 shared-epitope alleles and exposed to smoking. Clinical studies have identified associations among immune responses to *P. gingivalis*, ACPA-positive RA, smoking, and HLA risk alleles, although heterogeneous and sometimes negative findings require cautious interpretation [18,19,20]. *Aggregatibacter actinomycetemcomitans* may provide a complementary route through leukotoxin A-mediated hypercitrullination of host neutrophils [21]. Accordingly, periodontitis is presented here not as a single proven cause of RA, but as a tractable mucosal setting in which dysbiosis, post-translational protein modification, cytokine signaling, and genetic susceptibility converge.

Mechanical biofilm disruption and control of modifiable risk factors remain the standard of care for periodontitis and are effective in many patients. However, residual inflammation or recurrence may persist in susceptible individuals. This unmet need provides a rationale for investigating host-modulation strategies, although systemic biologics are not currently indicated for routine periodontal treatment [14,16].

Given the limitations and adverse effects associated with long-term administration of steroidal and non-steroidal anti-inflammatory drugs, biological therapies have attracted considerable attention from both the scientific community and the pharmaceutical industry. Their ability to selectively modulate molecular targets involved in inflammatory signaling pathways offers promising opportunities for the treatment of chronic inflammatory diseases [7,8,11].

### Literature Search Strategy

A systematic search of electronic databases, including PubMed, MEDLINE, Web of Science, Embase, and ClinicalTrials.gov, was performed. The literature indexed from database inception through May 2025 was reviewed. The search strategy incorporated the following keywords and Medical Subject Headings (MeSH) terms: “biologics”, “biopharmaceuticals”, “JAK inhibitors”, “Janus kinase inhibitors”, “STAT inhibitors”, “interleukin inhibitors”, “anti-TNF”, “B-cell inhibitors”, “plant-derived compounds”, “natural products”, and “natural derivatives”. Titles and abstracts identified through the search were screened for relevance.

No restrictions regarding publication date were applied. Studies were considered eligible irrespective of methodological design. Articles published in English that reported outcomes related to the investigated variables were included. In addition, the reference lists of all eligible publications were manually screened to identify potentially relevant studies not captured during the initial database search.

## 2. Types of Biological Therapies and Their Clinical Applications

The identification of cytokines as key mediators of inflammatory processes involved in the pathogenesis of rheumatic diseases has led to the development of biological therapies directed against specific cytokine signaling pathways. Currently approved cytokine inhibitors include agents targeting tumor necrosis factor-α (TNF-α), the interleukin-6 receptor (IL-6R), interleukin-1 (IL-1), interleukin-17 (IL-17), and the interleukin-12/23 axis [10,22,23,24,25].

**Figure 1:**
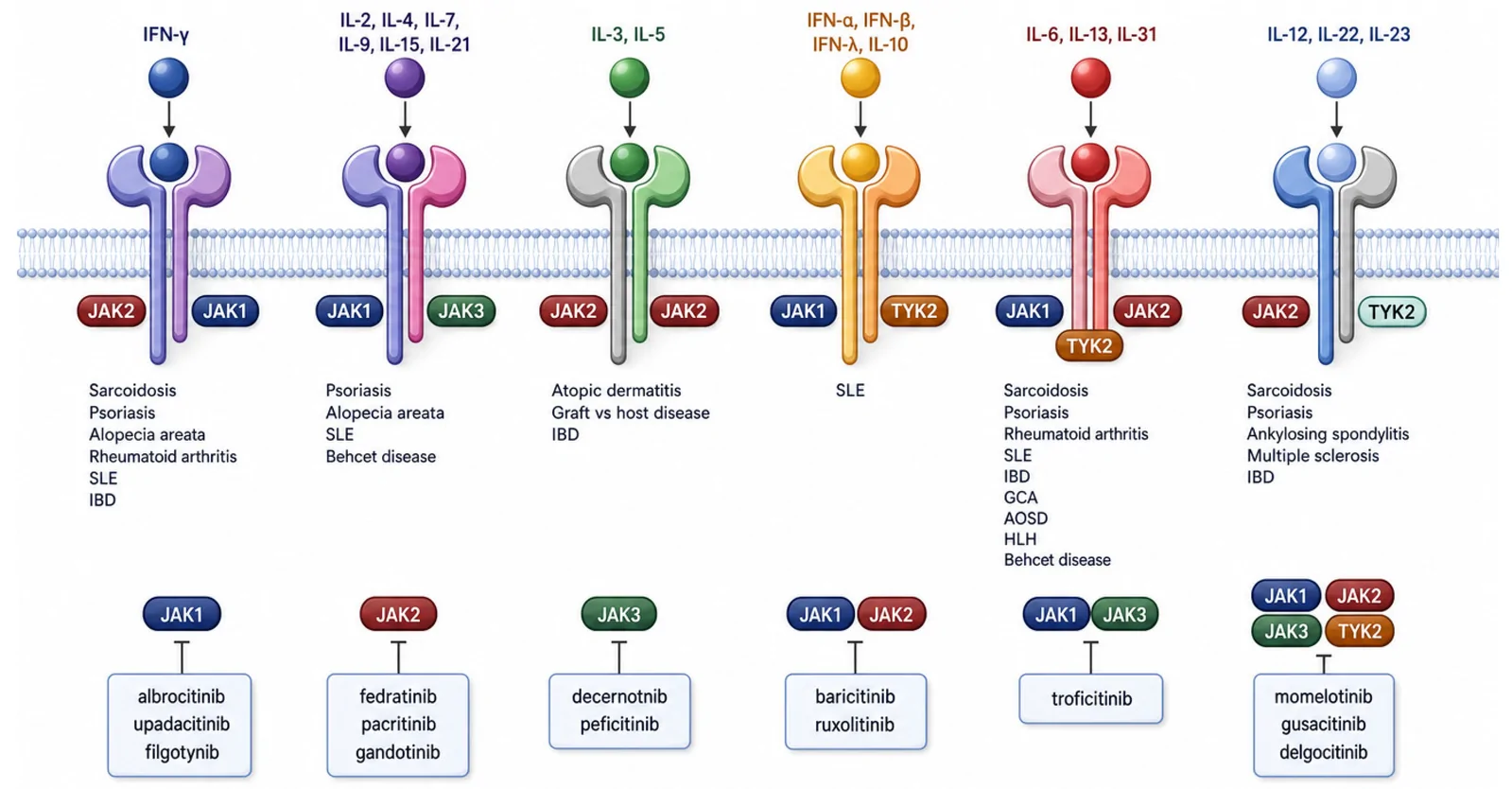
Cytokine-induced activation of JAKs triggers phosphorylation and dimerization of STAT proteins, followed by their nuclear translocation and regulation of genes involved in inflammation, immune responses, tissue remodeling, and bone metabolism. Pharmacological inhibition may be achieved through JAK inhibitors or STAT-targeting strategies, including peptide inhibitors, small interfering RNA (siRNA), and antisense oligonucleotides (ASOs). P, phosphate group. Created in BioRender. Brozek, R. (2026) https://BioRender.com/ui2dtha [11,26,27,28].

**Figure 2:**
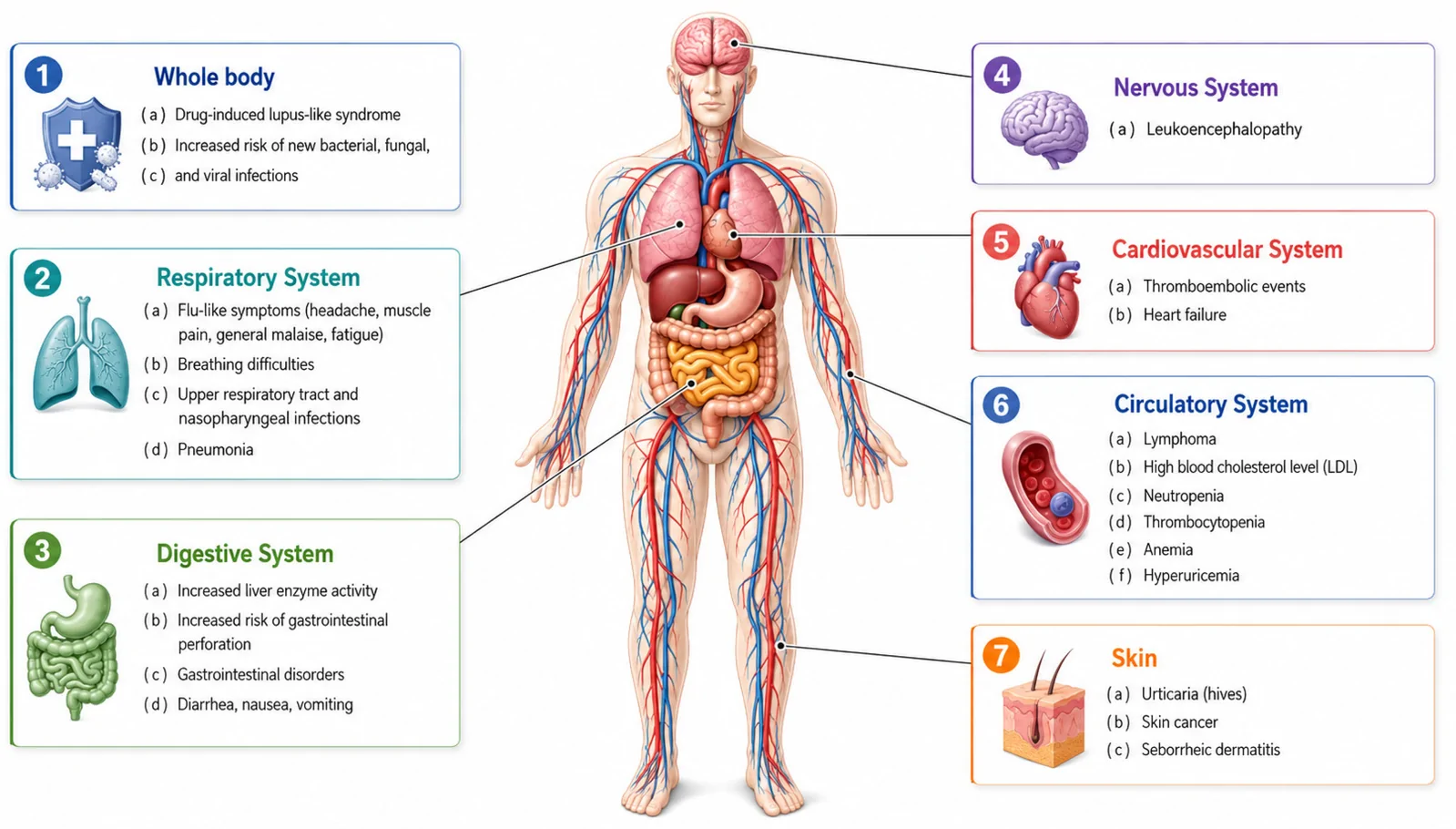
Systemic adverse effects associated with JAK inhibitor therapy. Pharmacological inhibition of the JAK/STAT signaling pathway may affect multiple organ systems due to its central role in immune regulation and cytokine signaling. Reported adverse events include increased susceptibility to infections, respiratory and gastrointestinal complications, thromboembolic and cardiovascular events, hematological abnormalities, neurological manifestations, and dermatological disorders. Careful patient monitoring is therefore required during long-term treatment with JAK inhibitors [29,30].

Figure 1 illustrates the cytokine receptor-associated JAK/STAT signaling pathways and the specific JAK family members involved in cytokine signal transduction. It also presents selected autoimmune and inflammatory diseases associated with these pathways, together with representative JAK inhibitors targeting individual kinase isoforms. The principal adverse effects associated with these therapeutic agents are summarized in Figure 2.

### 2.1. Tumor Necrosis Factor-Alpha Inhibitors (Anti-TNF-α Agents)

Tumor necrosis factor-alpha (TNF-α) inhibitors are established therapies for immune-mediated inflammatory diseases and include adalimumab, certolizumab pegol, etanercept, golimumab, and infliximab. Their principal class risks include serious or opportunistic infection and reactivation of latent tuberculosis. Additional safety concerns include demyelinating disease and worsening moderate-to-severe heart failure, whereas evidence concerning malignancy varies by cancer type and background risk [2,31,32].

TNF-α is a key regulator of inflammatory and immune responses and represents one of the most important pro-inflammatory cytokines involved in the pathogenesis of chronic inflammatory diseases. It exists in both a soluble form (soluble TNF-α; sTNF) and a membrane-bound form (transmembrane TNF-α; tmTNF). The biological activity of TNF-α is mediated through interaction with two distinct receptors, TNFR1 and TNFR2 (tumor necrosis factor receptors 1 and 2). Activation of these receptors initiates multiple intracellular signaling pathways that regulate immune and inflammatory processes. The pleiotropic effects of TNF-α include stimulation of pro-inflammatory cytokine production, particularly interleukin (IL)-1 and IL-6, upregulation of endothelial adhesion molecules, recruitment of neutrophils and monocytes to sites of inflammation, and activation of fibroblasts and osteoclasts. Excessive or dysregulated TNF-α signaling contributes to the maintenance of chronic inflammation, progressive tissue damage, and the development of clinical manifestations characteristic of autoimmune and inflammatory disorders [33].

The therapeutic activity of TNF-α inhibitors is based on their ability to bind circulating and/or membrane-bound TNF-α, thereby preventing its interaction with TNFR1 and TNFR2 expressed on target cells. Inhibition of receptor activation suppresses downstream pro-inflammatory signaling pathways, including cytokine- and chemokine-mediated responses involving IL-1 and IL-6. As a consequence, leukocyte recruitment to inflamed tissues is reduced through decreased expression of endothelial adhesion molecules, while activation of macrophages, fibroblasts, and osteoclasts is attenuated. Collectively, these effects result in a reduction in inflammatory activity, alleviation of clinical symptoms, and inhibition of disease progression [34].

### 2.2. B-Cell Inhibitors

B-cell-targeted therapies include belimumab and rituximab. B-cell activating factor (BAFF; also termed BLyS or TNFSF13B), a member of the TNF ligand superfamily, regulates B-cell survival, maturation, and differentiation through BAFF-R, TACI, and BCMA. In particular, BAFF-R preferentially activates the non-canonical NF-κB pathway, linking BAFF signaling to the maintenance of mature and autoreactive B-cell populations [35].

Belimumab is a fully human monoclonal antibody that neutralizes soluble BAFF and is approved for systemic lupus erythematosus and lupus nephritis. In contrast, rituximab targets CD20 on mature B cells and induces their depletion; it is used in rheumatoid arthritis when conventional DMARDs or TNF inhibitors provide an inadequate response [22,36].

B-cell-directed therapies increase susceptibility to infection. Rituximab is associated with hepatitis B virus reactivation and rare progressive multifocal leukoencephalopathy, whereas belimumab may cause infusion reactions, leukopenia, and serious infections; appropriate screening and monitoring are therefore required [36,37].

### 2.3. Interleukin Inhibitors

These therapeutic agents selectively target various pro-inflammatory interleukins involved in the pathogenesis of chronic inflammatory and autoimmune diseases. They have been widely utilized in the treatment of psoriatic arthritis, psoriasis, rheumatoid arthritis, and ankylosing spondylitis. The most commonly reported adverse events include hepatic dysfunction, upper respiratory tract infections, sinusitis, hypertension, injection-site reactions, nausea, and headache. Furthermore, treatment with interleukin inhibitors may be associated with serious adverse outcomes, including an increased susceptibility to severe infections and opportunistic infections, such as herpes zoster, tuberculosis, and toxoplasmosis. Long-term administration has also raised concerns regarding a potential increase in the risk of malignancies, although the available evidence remains inconclusive and requires further investigation [2].

#### 2.3.1. Interleukin-6 Inhibitors

Interleukin-6 (IL-6) plays a pivotal role in maintaining cellular homeostasis and contributes to host defense against infections and various stressors. However, dysregulated IL-6 production may result in excessive inflammatory responses and contribute to the pathogenesis of numerous inflammatory and autoimmune diseases. Furthermore, IL-6 is critically involved in the regulation and differentiation of T helper cell subsets, particularly in maintaining the balance between regulatory T cells (Tregs) and T helper 17 (Th17) cells. Currently, two IL-6 receptor antagonists are approved for clinical use: sarilumab and tocilizumab. Reported adverse effects of tocilizumab include an increased risk of gastrointestinal perforation, skin infections, dyslipidemia, and hepatic dysfunction [23,38].

IL-6 inhibitors exert their therapeutic effects by blocking both membrane-bound and soluble IL-6 receptors, thereby preventing IL-6-mediated signal transduction and suppressing inflammatory processes. Consequently, several biological functions regulated by IL-6, including cellular proliferation, survival, acute-phase protein synthesis, and immune activation, are inhibited [9].

#### 2.3.2. Interleukin-1 Inhibitors

Interleukin-1 is a central mediator of innate inflammation, and excessive IL-1 signaling contributes to autoinflammatory and immune-mediated disease. IL-1 blockade has established roles in several autoinflammatory syndromes and Still’s disease, although efficacy varies by disease context [10].

Identification of the endogenous IL-1 receptor antagonist enabled development of anakinra. Anakinra competitively blocks IL-1R1, canakinumab neutralizes IL-1β, and rilonacept acts as a soluble decoy receptor. These agents are used for selected autoinflammatory disorders, with anakinra also approved for rheumatoid arthritis [10].

#### 2.3.3. Interleukin-17 Inhibitors

Overexpression of interleukin-17 (IL-17) promotes activation of multiple inflammatory pathways and induces the production of several pro-inflammatory cytokines, including IL-1β, IL-6, IL-8, and tumor necrosis factor-α (TNF-α). Consequently, IL-17 has emerged as an important therapeutic target in chronic inflammatory diseases [12,24,39].

IL-17 inhibitors are used principally for psoriasis, psoriatic arthritis, and axial spondyloarthritis. Secukinumab and ixekizumab neutralize IL-17A; class-specific safety considerations include mucocutaneous candidiasis and other infections [24,39].

#### 2.3.4. Interleukin-12 and Interleukin-23 Inhibitors

Interleukin-12 (IL-12) and interleukin-23 (IL-23) belong to the IL-12 cytokine family and share a common p40 protein subunit. IL-12 primarily promotes Th1 differentiation, whereas IL-23 supports maintenance and expansion of Th17 responses [25].

Inhibitors targeting IL-12 and IL-23 are widely used in the management of psoriasis and psoriatic arthritis. Ustekinumab, a fully human IgG1κ monoclonal antibody directed against the shared p40 subunit, effectively neutralizes the biological activity of both IL-12 and IL-23 and has demonstrated significant clinical efficacy in these conditions [25].

### 2.4. Selective Co-Stimulatory Modulators

Abatacept is currently the only approved representative of the class of selective co-stimulation modulators. It is indicated for the treatment of moderate-to-severe rheumatoid arthritis, juvenile idiopathic arthritis, and psoriatic arthritis in patients who exhibit an inadequate response to methotrexate or other conventional disease-modifying antirheumatic drugs (DMARDs). Its mechanism of action is based on the inhibition of T-cell activation through selective modulation of co-stimulatory signaling pathways. The most commonly reported adverse effects include headache, upper respiratory tract symptoms, sore throat, and nausea. Similar to other biological therapies, abatacept is also associated with an increased risk of infections and, potentially, malignancies [40].

### 2.5. JAK Inhibitors

The therapeutic efficacy of Janus kinase (JAK) inhibitors is based on their ability to suppress cytokine-mediated intracellular signaling pathways. JAKs are involved in signal transduction initiated by several cytokines that play critical roles in immune regulation, including interleukin (IL)-2, IL-6, IL-12, IL-23, and interferons (IFNs). However, the activity of JAK inhibitors may be regarded as a double-edged sword. While inhibition of a specific JAK isoform can effectively attenuate pathological inflammatory responses, it may also interfere with other signaling pathways, potentially leading to undesirable adverse effects [11,29,30].

The selectivity of JAK inhibitors toward individual JAK isoforms depends on several factors, including drug concentration, target cell type, receptor expression patterns, and pharmacokinetic properties [11].

- JAK/STAT Pathway: Structure, Function, and Signal Transduction

The Janus kinase/signal transducer and activator of transcription (JAK/STAT) pathway is one of the principal signaling mechanisms responsible for transmitting extracellular cytokine and growth factor signals to the cell nucleus. This pathway comprises cell-surface cytokine and growth factor receptors, cytoplasmic JAKs, and members of the STAT family of transcription factors [26].

Activation of the JAK/STAT pathway is initiated when a cytokine binds to its cognate receptor on the cell surface. This interaction triggers intracellular signal transduction events that ultimately lead to the activation of gene transcription within the nucleus, resulting in the synthesis of proteins required for an appropriate cellular response [11,26].

Four members of the JAK family have been identified: JAK1, JAK2, JAK3, and tyrosine kinase 2 (TYK2). Upon receptor activation, conformational changes facilitate JAK activation and subsequent phosphorylation of STAT proteins associated with the receptor complex. STAT proteins function as transcription factors that regulate gene expression. To date, seven STAT family members have been identified: STAT1, STAT2, STAT3, STAT4, STAT5A, STAT5B, and STAT6. Following phosphorylation, STAT proteins form homo- or heterodimers and translocate to the nucleus, where they bind to specific regulatory DNA sequences and modulate the transcription of target genes involved in cellular proliferation, differentiation, survival, apoptosis, and immune responses [41,42].

- Classical JAK/STAT Signaling Mechanism

In the classical JAK/STAT signaling pathway, cytokine binding induces receptor dimerization, bringing receptor-associated JAK molecules into close proximity. This process promotes JAK transphosphorylation and activation. Activated JAKs subsequently phosphorylate tyrosine residues within the cytoplasmic domain of the receptor, creating docking sites for STAT proteins. Once recruited to the receptor complex, STAT proteins are phosphorylated by JAKs [11,26].

**Figure 3:**
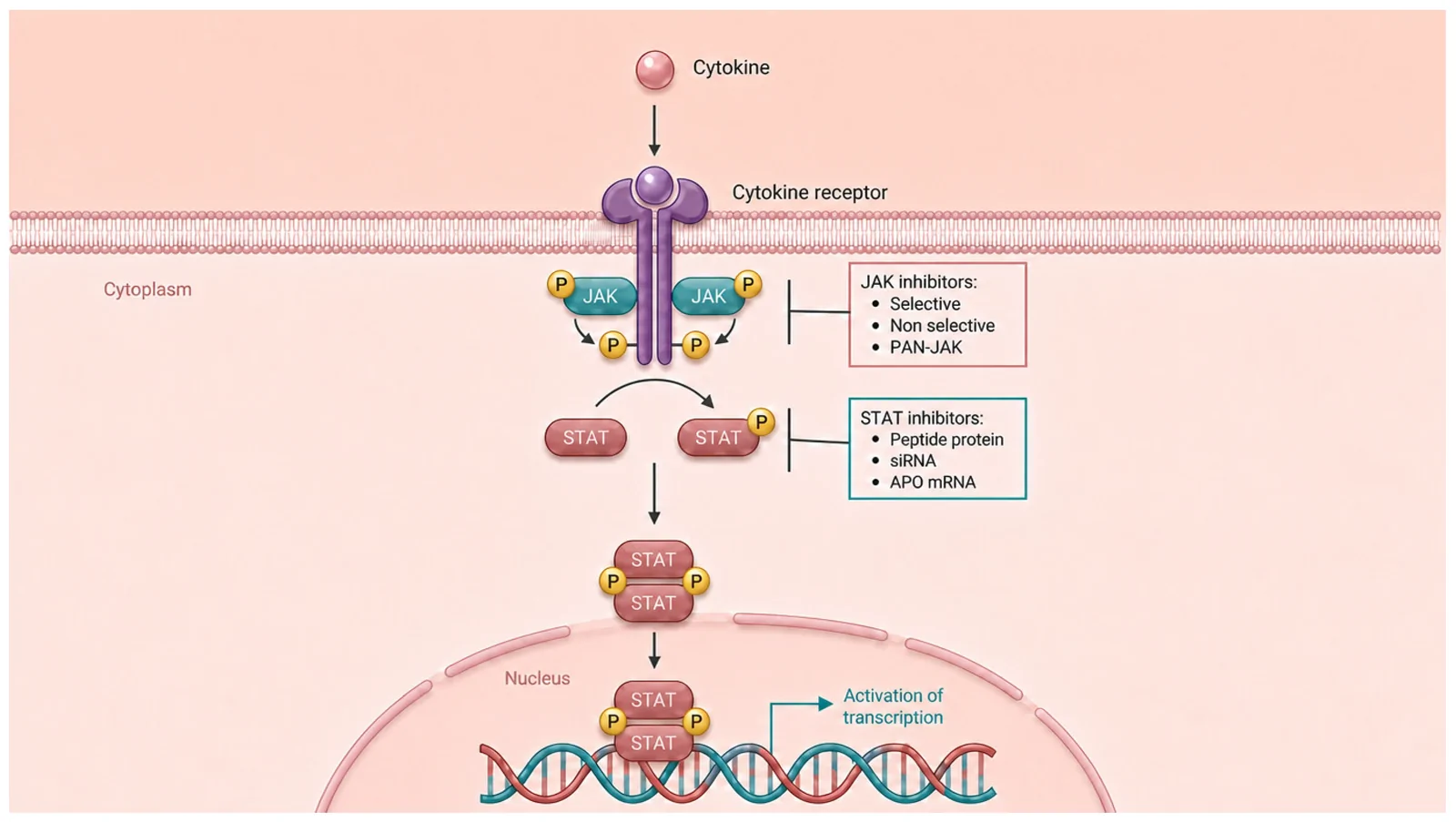
Functional organization of cytokine receptor-associated JAK signaling complexes and pharmacological targeting of JAK family kinases. Binding of cytokines to their cognate receptors induces activation of specific JAK family members (JAK1, JAK2, JAK3, and TYK2), leading to downstream STAT signaling and transcriptional regulation of genes involved in immune homeostasis and inflammatory responses. Distinct cytokine receptor complexes utilize characteristic JAK combinations, thereby determining the specificity of intracellular signaling and disease-associated immune pathways. Aberrant activation of JAK/STAT signaling contributes to the development of autoimmune, autoinflammatory, and chronic inflammatory disorders. Therapeutic inhibition of JAKs may be achieved using selective inhibitors targeting individual JAK isoforms or broader inhibitors targeting multiple JAK family members. Representative JAK inhibitors and their selectivity profiles are shown in the lower panel. Phosphorylation of JAK and STAT proteins is indicated by the yellow circles marked “P”. Created in BioRender. Brozek, R. (2026) https://BioRender.com/a8noug5 [11,26].

Phosphorylated STAT molecules then dissociate from the receptor and form homo- or heterodimers, which translocate to the nucleus. There, they bind to promoter or enhancer regions of target genes and regulate transcriptional activity [11,43]. A schematic representation of this signaling mechanism is presented in Figure 3.

Several mechanisms of STAT-mediated transcriptional regulation have been described. In the canonical mechanism, STAT dimers directly bind specific DNA sequences within the promoters of target genes. Alternatively, STAT proteins may form transcriptional complexes with other transcription factors, thereby modulating their activity. STAT proteins may also interact with non-STAT regulatory elements to facilitate STAT-dependent transcription. Furthermore, transcription factors unrelated to the STAT family may cooperate with STAT proteins by binding distinct regulatory regions of DNA, thereby contributing to the regulation of gene expression [11,41].

- Non-Canonical JAK/STAT Signaling and Exceptions to the Classical Model

The classical model does not encompass the full range of JAK and STAT activities. In non-canonical signaling, STAT proteins may regulate gene expression without phosphorylation of the conserved tyrosine residue or formation of conventional dimers. Unphosphorylated STATs (U-STATs) shuttle between the cytoplasm and nucleus and cooperate with other transcription factors. For example, U-STAT3 forms a complex with NF-κB that sustains the expression of selected inflammatory genes after IL-6 stimulation, whereas STAT2–IRF9 complexes contribute to the basal expression of interferon-stimulated genes without full activation of the classical ISGF3 complex [44,45].

JAK and STAT proteins also exert non-transcriptional and epigenetic functions. Nuclear JAK2 can directly phosphorylate histone H3 at Tyr41, leading to displacement of heterochromatin protein 1 alpha (HP1α) and altered chromatin accessibility. Mitochondrial STAT3 regulates respiratory-chain activity, cellular metabolism, and reactive oxygen species production independently of its classical activity as a nuclear transcription factor [46,47]. These mechanisms link cytokine signaling to the metabolic and epigenetic state of the cell and may influence the duration of inflammatory responses.

Exceptions also occur at individual stages of the classical model. Some cytokine receptors, including the growth hormone receptor, exist as inactive dimers before ligand binding; activation therefore results from a conformational rearrangement of a preformed complex rather than receptor dimerization. Moreover, STAT proteins can be activated independently of JAKs by other tyrosine kinases, including members of the Src family such as Lyn. STAT2 is another exception because, in type I interferon signaling, it acts predominantly within the heterotrimeric STAT1–STAT2–IRF9 complex rather than as a conventional STAT homodimer [45,48,49].

The JAK/STAT pathway is used primarily by cytokines that signal through type I and type II receptors, including interferons, many interleukins, and hematopoietic growth factors. Other cytokines, such as IL-1, IL-17, and TNF-α, signal mainly through distinct intracellular systems; for example, IL-1 and IL-17 principally engage the MyD88–IRAK–TRAF6 and Act1–TRAF6 axes, respectively, to activate NF-κB and mitogen-activated protein kinases [12,50]. Nevertheless, extensive crosstalk with NF-κB, PI3K/AKT, and MAPK pathways makes the final cellular response dependent on the cytokine, receptor, cell type, and timing and intensity of stimulation. Consequently, measurement of phosphorylated STAT proteins or inhibition of JAK activity may not capture all non-canonical and JAK-independent functions of this network.

According to their molecular targets and spectrum of activity, currently available inhibitors used in research and clinical practice can be classified into the following categories:

#### 2.5.1. First-Generation Inhibitors

JAK inhibitors are designed to suppress the enzymatic activity of Janus kinases, thereby attenuating intracellular signal transduction mediated through the JAK/STAT pathway. By inhibiting JAK-dependent signaling, these agents reduce the production and activity of pro-inflammatory cytokines, the expression of which is frequently dysregulated in chronic inflammatory and autoimmune disorders. Consequently, JAK inhibitors exert immunomodulatory and immunosuppressive effects. Unlike monoclonal antibodies, JAK inhibitors are small-molecule compounds capable of penetrating the cell membrane and interacting directly with intracellular kinases, thereby preventing downstream signal propagation [11].

First-generation JAK inhibitors act as adenosine triphosphate (ATP)-competitive inhibitors by binding to the catalytic ATP-binding pocket within the kinase domain. The tyrosine kinase domain exhibits a highly conserved ATP-binding site in its active conformation. This structural feature has facilitated the development of compounds targeting JAKs, particularly JAK1. However, because the ATP-binding pocket is highly conserved among members of the JAK family, first-generation inhibitors generally exhibit limited selectivity and may inhibit multiple JAK isoforms simultaneously. As a result, these compounds are commonly classified as pan-JAK inhibitors [11].

The first generation of JAK inhibitors includes the following agents:

Baricitinib is a selective inhibitor of JAK1 and JAK2, with lower inhibitory activity against JAK3 and tyrosine kinase 2 (TYK2). It has been approved for the treatment of rheumatoid arthritis and atopic dermatitis and has also demonstrated therapeutic potential in juvenile dermatomyositis, systemic lupus erythematosus, and interferon-mediated autoinflammatory disorders. Its clinical efficacy is primarily associated with suppression of inflammatory signaling and prevention of disease progression. The most frequently reported adverse effects are dose-dependent and include an increased risk of infections, particularly herpes zoster and tuberculosis, as well as elevations in serum lipid concentrations. Special caution is recommended in patients with pre-existing cardiovascular risk factors [51].

Ruxolitinib is a potent inhibitor of JAK1 and JAK2. It was initially approved in North America for the treatment of myelofibrosis (MF) and subsequently received approval in Europe for the management of polycythemia vera. In patients with MF, its therapeutic effects are primarily attributed to suppression of aberrant JAK/STAT pathway activation and reduction in pathological myeloproliferative activity. Consequently, ruxolitinib effectively alleviates disease-related symptoms and improves clinical outcomes [52].

Tofacitinib preferentially inhibits JAK1 and JAK3, with weaker activity against JAK2 and TYK2. It is approved for several immune-mediated diseases, including rheumatoid arthritis, psoriatic arthritis, ankylosing spondylitis, and ulcerative colitis, depending on jurisdiction. Major safety concerns include serious infection, herpes zoster, malignancy, venous thromboembolism, and major adverse cardiovascular events in susceptible patients [29,30,53].

#### 2.5.2. Second-Generation Inhibitors

Recent advances in the development of highly selective kinase-targeting compounds have led to the emergence of a new generation of therapeutic agents, including the following:

#### JAK1 Inhibitors

Filgotinib is a preferential JAK1 inhibitor that suppresses JAK1-dependent cytokine signaling. In Europe, it is approved for moderate-to-severe rheumatoid arthritis after inadequate response or intolerance to disease-modifying antirheumatic drugs. In the FINCH 2 trial, filgotinib improved clinical response in patients with refractory rheumatoid arthritis; commonly reported events included upper respiratory tract infection and nasopharyngitis [54].

Upadacitinib is a selective JAK1 inhibitor that effectively suppresses signaling mediated by JAK1-dependent cytokines, including IL-6, IL-2, and interferon-gamma (IFN-γ). It is currently approved for the treatment of rheumatoid arthritis in patients with an inadequate response or intolerance to methotrexate. Ongoing clinical studies are investigating its therapeutic potential in additional immune-mediated disorders, including ulcerative colitis, atopic dermatitis, Crohn’s disease, psoriatic arthritis, and ankylosing spondylitis. Frequently reported adverse effects include elevations in hepatic aminotransferases, serum lipid levels, and creatine phosphokinase activity. Serious adverse events may include venous thromboembolism, cerebrovascular events, and, in rare cases, death [55].

Abrocitinib is a selective JAK1 inhibitor approved for the treatment of moderate-to-severe atopic dermatitis. By selectively targeting JAK1-mediated signaling pathways, it modulates cytokine-driven inflammatory responses associated with disease pathogenesis. The most frequently reported adverse events include nausea, diarrhea, headache, upper respiratory tract infections, nasopharyngitis, and hematological abnormalities [56].

#### JAK2 Inhibitors

Fedratinib is a potent and selective Janus kinase 2 (JAK2) inhibitor that competitively binds to the ATP-binding site of the enzyme. The drug has been approved in the United States for the treatment of primary and secondary myelofibrosis and has received marketing authorization in Europe for patients with splenomegaly associated with primary myelofibrosis. Fedratinib exerts antineoplastic activity by promoting apoptosis of malignant cells. However, its use has been associated with serious adverse events, including severe and potentially fatal encephalopathies, particularly Wernicke’s encephalopathy. The most frequently reported adverse effects include nausea, diarrhea, anemia, vomiting, and thrombocytopenia [57].

Pacritinib is an orally administered kinase inhibitor developed for myelofibrosis, including patients with baseline cytopenias. In the phase III PERSIST-1 trial, gastrointestinal and hematological adverse events were among the principal treatment limitations [58].

Gandotinib is a selective JAK2 inhibitor developed for the treatment of chronic myeloproliferative neoplasms (MPNs), including polycythemia vera and essential thrombocythemia. Preclinical studies demonstrated a significant reduction in the number of Ba/F3 JAK2-positive cells within the spleens of immunodeficient mice. Furthermore, gandotinib effectively inhibited signaling mediated by the JAK2 V617F mutation while exerting minimal effects on cells dependent on wild-type JAK2 signaling [59]. Clinical studies have demonstrated its efficacy in patients with JAK2 V617F-positive MPNs, including those previously treated with ruxolitinib. A phase II clinical trial evaluated gandotinib in patients with myelofibrosis, polycythemia vera, and essential thrombocythemia. The most frequently reported adverse events included anemia, hyperuricemia, diarrhea, thrombocytopenia, and fatigue. Notably, in patients with myelofibrosis previously treated with ruxolitinib, gandotinib did not exhibit significant hematological or neurological toxicity during phase II evaluation [60].

#### JAK3 Inhibitors

Decernotinib is a selective JAK3 inhibitor. In preclinical studies, it significantly reduced ankle edema in an animal model of T-cell-dependent inflammatory skin disease [61]. Phase II clinical trials demonstrated its efficacy in alleviating the symptoms of rheumatoid arthritis. Reported adverse effects included lymphopenia, neutropenia, and elevated serum aminotransferase levels [62].

Peficitinib is a JAK inhibitor with preferential activity toward JAK3, capable of suppressing T-cell proliferation and inhibiting STAT5 phosphorylation. The drug has been approved in Japan for the treatment of rheumatoid arthritis. In addition, its therapeutic potential is being investigated in other immune-mediated disorders, including ulcerative colitis and psoriasis. The most commonly reported adverse events include nasopharyngitis, elevated plasma creatine kinase levels, pneumonia, bronchitis, herpes zoster, and cystitis. Serious adverse events, such as sepsis and gastrointestinal perforation, have also been reported [63].

#### Pan-JAK Inhibitors

Momelotinib is a selective ATP-competitive inhibitor of JAK1 and JAK2 kinases. Clinical studies have demonstrated its efficacy in the treatment of myelofibrosis (MF). The most commonly reported adverse events include cough, nausea, diarrhoea, anaemia, thrombocytopenia, neutropenia, and peripheral neuropathy [64].

Gusacitinib is a multi-target kinase inhibitor that acts on JAK1, JAK2, JAK3, and TYK2, while also inhibiting spleen tyrosine kinase (SYK). Through modulation of both Th1- and Th2-mediated inflammatory pathways, gusacitinib has demonstrated therapeutic potential in chronic hand eczema [65]. The drug is currently being investigated for the treatment of chronic hand eczema and basal cell carcinoma. In addition, Phase I clinical trials are evaluating its safety and efficacy in patients with systemic lupus erythematosus [66].

Delgocitinib is a pan-JAK inhibitor targeting all members of the JAK family. It has been approved in Japan as a topical formulation for the treatment of atopic dermatitis. Clinical studies have also demonstrated its efficacy in atopic dermatitis, chronic hand eczema, and alopecia areata. The most frequently reported adverse effects include contact dermatitis, nasopharyngitis, and acne, whereas Kaposi’s varicelliform eruption has been reported as a rare but serious adverse event [67].

### 2.6. STAT Inhibitors

The development of STAT inhibitors has primarily focused on preventing STAT phosphorylation and dimerization, two critical steps required for downstream signal transduction and transcriptional activation. Direct inhibitory strategies have targeted the Src homology 2 (SH2) domain, the DNA-binding domain, and the N-terminal region of STAT3. Peptide- and peptidomimetic-based inhibitors have been designed to disrupt STAT3 dimer formation and thereby suppress its transcriptional activity. However, their clinical applicability remains limited owing to poor cellular permeability and insufficient molecular stability [27,28].

Alternative approaches include STAT3 decoy oligonucleotides and small interfering RNA (siRNA)-based therapeutics, which have demonstrated the potential to selectively inhibit STAT3 signaling. Nevertheless, their therapeutic utility is constrained by challenges related to stability and efficient delivery [27,28].

Non-peptide small molecules and oligonucleotide-based compounds can interfere with STAT DNA binding or reduce STAT3 expression. Antisense oligonucleotides and siRNA therefore offer target selectivity but remain constrained by stability and delivery. Curcumin and resveratrol also modulate STAT3 and interconnected inflammatory pathways, although their effects are pleiotropic [28,68,69].

For clarity and to facilitate comparison with the natural compounds discussed below, the principal molecular targets, representative agents, mechanisms of action, clinical applications, and major limitations of conventional anti-inflammatory therapies are summarized in Table 1.

### 2.7. The Role of the JAK/STAT Signaling Pathway in Periodontitis

Accumulating evidence suggests that dysregulation of JAK–STAT signaling may play a significant role in the pathogenesis of periodontitis. Reduced levels of interleukin-10 (IL-10) have been shown to impair STAT3 and STAT5 activity in murine models, whereas elevated concentrations of IL-12 and IL-1α promote the differentiation of T helper (Th) cells toward the Th1 phenotype. Through the enhancement of cell-mediated immune responses, Th1 lymphocytes contribute to the exacerbation of periodontal tissue damage induced by *Porphyromonas gingivalis* infection [13,70].

Proteins belonging to the suppressor of cytokine signaling (SOCS) family may contribute to the inhibition of periodontitis progression through the modulation of immunocompetent cell functions and regulation of anti-inflammatory immune responses. SOCS proteins constitute a family of intracellular negative regulators of cytokine signaling that control immune responses mediated through the Janus kinase/signal transducer and activator of transcription (JAK/STAT) pathway. Their biological activity is closely associated with signaling induced by cytokines, interferons, and growth factors. Acting through a negative feedback mechanism, SOCS proteins interact with cytokine receptors and cytoplasmic JAKs, thereby preventing STAT recruitment and activation and ultimately suppressing downstream signal transduction [43,71].

A study conducted by Papathanasiou et al. [71] using SOCS-3-deficient knockout (KO) mice demonstrated significantly greater alveolar bone loss following *P. gingivalis* infection compared with wild-type controls. SOCS-3 deficiency was additionally associated with increased osteoclast numbers and elevated expression of receptor activator of nuclear factor κB ligand (RANKL). Furthermore, induction of inflammation by administration of *P. gingivalis* lipopolysaccharide resulted in enhanced secretion of the pro-inflammatory cytokines IL-1β, IL-6, and IL-8 in SOCS-3-deficient animals. These findings indicate that SOCS-3 plays an important protective role in regulating inflammatory bone resorption during periodontitis [71].

**Figure 4:**
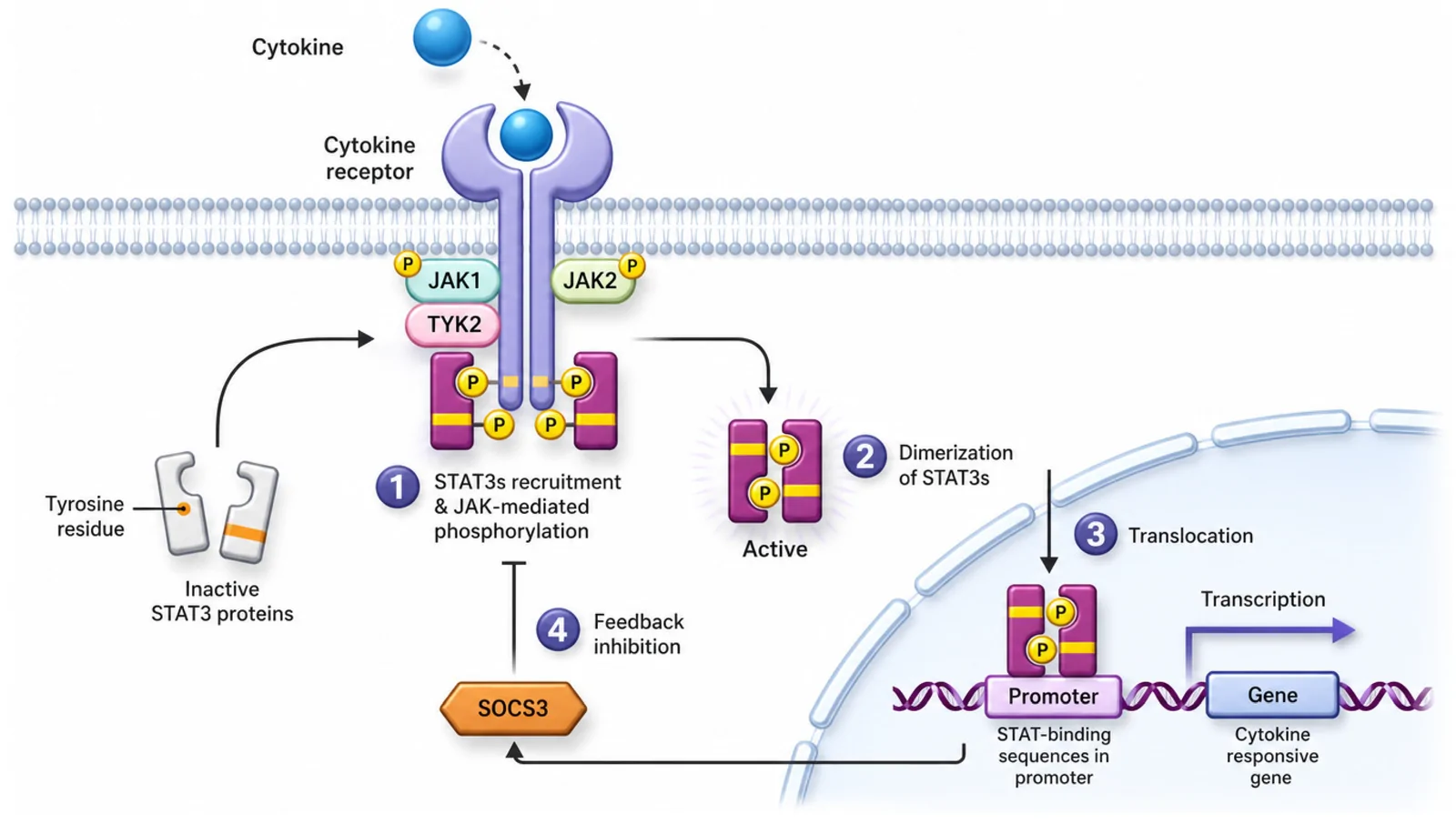
Schematic representation of the JAK/STAT3 signaling pathway and SOCS3-mediated negative feedback regulation. Cytokine binding activates receptor-associated JAKs, leading to STAT3 recruitment, phosphorylation, dimerization, and nuclear translocation. Activated STAT3 dimers bind promoter regions of target genes and initiate transcription of cytokine-responsive genes. Induction of suppressor of cytokine signaling 3 (SOCS3) provides a negative feedback mechanism that inhibits JAK activity and limits excessive STAT3 signaling, thereby regulating inflammatory and immune responses. The dashed arrow indicates cytokine binding to its receptor and initiation of JAK/STAT signaling. Subsequent arrows represent STAT3 phosphorylation, dimerization, nuclear translocation, and activation of target-gene transcription, including SOCS3. The terminal inhibitory symbol indicates SOCS3-mediated negative feedback, which suppresses further JAK/STAT signaling. Created in BioRender. Brozek, R. (2026) https://BioRender.com/7qp7hzu [43,71].

Binding of SOCS proteins to cytokine receptors alters receptor-associated signaling complexes and interferes with STAT activation, thereby modulating the activity of the intracellular JAK/STAT signaling pathway. Through this mechanism, SOCS proteins contribute to the maintenance of immune homeostasis and regulation of inflammatory responses. Schematic representation of the JAK/STAT3 signaling pathway and SOCS3-mediated negative feedback regulation is illustrated in Figure 4 [43,71].

In a 24-week prospective study of patients with active rheumatoid arthritis, baricitinib treatment was associated with improvement in periodontal inflammatory indices; however, the small, uncontrolled design precludes causal inference [72]. Two case reports likewise described favorable periodontal changes during tofacitinib therapy [73]. These observations are hypothesis-generating and do not establish a periodontal indication for systemic JAK inhibitors.

In vitro studies have shown that IL-6 and IL-11 can enhance alkaline phosphatase activity and osteogenic differentiation in human periodontal ligament cells. These findings link cytokine signaling to periodontal bone remodeling, but they do not establish clinical benefit from JAK inhibition [74,75].

A comprehensive understanding of the molecular mechanisms underlying JAK–STAT signaling may provide valuable insights into its regulatory functions and contribution to the pathogenesis of periodontitis. Further investigation is required to evaluate the therapeutic potential of agents targeting the JAK–STAT pathway. In combination with currently available periodontal therapies, modulation of host immune responses at the molecular level may represent a promising strategy for improving treatment outcomes and preventing disease progression [14,76].

### 2.8. Plant-Derived Compounds Targeting the JAK/STAT Signaling Pathway

Natural products are an important source of pharmacologically active small molecules. The compounds discussed here are not biological medicines; they are included as complementary probes of JAK/STAT and interconnected inflammatory pathways. To maintain clinical relevance and a focused evidence base, this section emphasizes artesunate, epigallocatechin-3-gallate (EGCG), celastrol, curcumin, and resveratrol [68,69,77,78,79].

Preclinical studies indicate that these compounds can reduce STAT3 activation and can also influence NF-κB, Nrf2, PI3K/AKT, oxidative stress, and apoptosis-related networks. Because the compounds are multitarget agents, pathway modulation observed in vitro should not be interpreted as evidence of selective JAK inhibition in patients [68,69,77,78,79].

Curcumin and resveratrol have the most direct, although still preliminary, human evidence in rheumatoid arthritis. Evidence for EGCG, celastrol, and artesunate in autoimmune disease remains preclinical, with mechanisms varying by model and compound [80,81,82,83,84].

Their pleiotropy may be useful in complex inflammatory disorders but also complicates target attribution, dose selection, and assessment of off-target effects. Consequently, changes in a STAT readout should be interpreted alongside parallel signaling and functional endpoints [68,69,77,78,79].

Clinical translation is limited by predominantly preclinical evidence and by compound-specific problems involving bioavailability, pharmacokinetics, selectivity, dose standardization, and safety. Formulation and delivery strategies may improve exposure, but they do not replace the need for controlled clinical trials [68,69,77,78,79].

Table 2 therefore summarizes the five representative compounds retained after evidence-focused selection, whereas Table 3 distinguishes preliminary clinical observations from preclinical autoimmune models [68,69,77,78,79,80,81,82,83,84].

The disease assignments in Table 3 indicate experimental or exploratory settings and do not imply approved indications. Among the listed compounds, human evidence in autoimmune disease remains limited to small studies of curcumin and resveratrol in RA [80,81]. Evidence for EGCG, celastrol, and artesunate is predominantly preclinical [82,83,84]. These findings extend the discussion beyond NF-κB to STAT3, Nrf2, PI3K/AKT/mTOR, BAFF-related, and T-cell differentiation networks, but they do not establish that JAK/STAT modulation is the dominant mechanism in patients. Accordingly, these products should be described as investigational adjuncts rather than alternatives to approved biologics or disease-modifying antirheumatic drugs.

#### Future Perspectives

Plant-derived modulators of JAK/STAT-related signaling remain investigational. Future studies should prioritize standardized formulations, pharmacokinetic characterization, target-engagement biomarkers, clinically relevant comparators, and adequately powered safety and efficacy endpoints before these compounds are considered therapeutic adjuncts [68,69,77,78,79,80,81,82,83,84].

### 2.9. NF-κB Signaling Pathway in Periodontitis

Nuclear factor kappa B (NF-κB) is a family of transcription factors that regulate the expression of numerous genes involved in inflammation, immune responses, cell proliferation, apoptosis, oxidative stress, and tissue remodeling. Dysregulated or persistent activation of NF-κB has been implicated in the pathogenesis of a wide range of inflammatory, autoimmune, and neoplastic diseases, including periodontitis. Given its central role in the regulation of inflammatory signaling, NF-κB has emerged as an attractive therapeutic target for the development of novel anti-inflammatory interventions [15,85].

Although inhibition of NF-κB signaling has been shown to attenuate inflammatory responses in experimental models, the precise contribution of this pathway to the initiation and progression of periodontitis remains incompletely understood. Currently, no therapeutic agents specifically targeting NF-κB have demonstrated sufficient efficacy to control periodontal disease progression in routine clinical practice. Consequently, further mechanistic and translational studies are required to clarify the therapeutic potential of NF-κB modulation in periodontal diseases [15].

#### 2.9.1. Mechanisms of NF-κB Activation

The NF-κB family derives its name from the nuclear factor initially identified in 1986 as a protein capable of binding to the enhancer sequence of the immunoglobulin κ light-chain gene in B lymphocytes. NF-κB proteins constitute essential regulators of inflammatory responses and bone remodeling processes. Their expression and activity are tightly controlled through multiple feedback mechanisms to ensure appropriate cellular responses to environmental stimuli [85].

**Figure 5:**
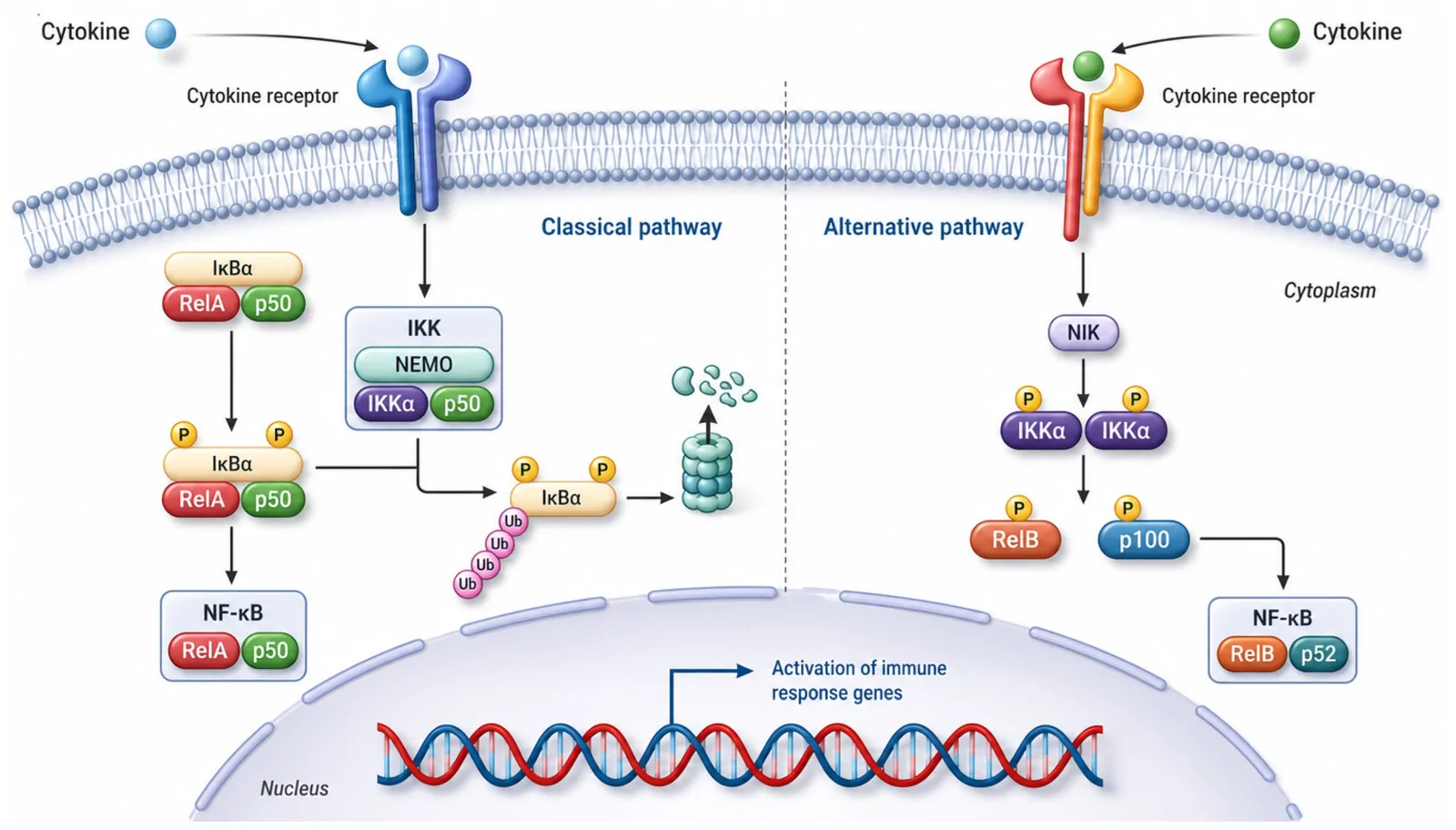
Classical (canonical) and alternative (non-canonical) NF-κB activation pathways. In the classical pathway, phosphorylation of inhibitor of κB (IκB) proteins by the IκB kinase (IKK) complex triggers their ubiquitination and subsequent proteasomal degradation. This process releases active NF-κB dimers, which translocate from the cytoplasm to the nucleus and regulate the transcription of target genes involved in inflammation and immune responses. In the non-canonical pathway, activation depends on the coordinated activity of NF-κB-inducing kinase (NIK) and IKKα. Activated IKKα phosphorylates the p100 precursor of the NF-κB2/RelB complex, leading to its proteolytic processing and generation of the mature p52/RelB heterodimer, which subsequently translocates to the nucleus and regulates the expression of genes involved in immune regulation and tissue homeostasis. The letter “P” in the yellow circles denotes phosphorylation, indicating the addition of phosphate groups to signaling proteins during NF-κB activation. The vertical dashed line separates the two distinct NF-κB activation routes: the classical (canonical) pathway on the left and the alternative (non-canonical) pathway on the right. Created in BioRender. Brozek, R. (2026) https://BioRender.com/sat8vty [15,85].

Cellular exposure to inflammatory mediators, microbial products, cytokines, and various stress-related factors activates intracellular signaling cascades that ultimately converge on NF-κB. Two major pathways regulate NF-κB activation: the canonical (classical) pathway and the non-canonical (alternative) pathway are presented in Figure 5 [15,85].

The canonical pathway is primarily activated by pro-inflammatory stimuli, including tumor necrosis factor-α (TNF-α), interleukin-1 (IL-1), and bacterial lipopolysaccharides (LPS). Activation of this pathway depends on phosphorylation of inhibitory κB proteins (IκBs) by the IκB kinase (IKK) complex, which consists of the catalytic subunits IKKα and IKKβ and the regulatory component NF-κB essential modulator (NEMO, also known as IKKγ). Following phosphorylation, IκB proteins undergo ubiquitination and proteasomal degradation, thereby releasing active NF-κB dimers. These dimers subsequently translocate to the nucleus, where they regulate the transcription of target genes involved in inflammation and immune responses [15,85].

In contrast to the rapid and usually transient activation of the canonical pathway, the non-canonical (alternative) NF-κB pathway is activated more slowly and produces a more sustained transcriptional response. It is triggered by selected members of the tumor necrosis factor receptor superfamily, including BAFF receptor (BAFF-R), CD40, lymphotoxin-β receptor (LTβR), and RANK. In unstimulated cells, NF-κB-inducing kinase (NIK) is constitutively targeted for degradation by the TRAF2-TRAF3-cIAP1/2 complex. Receptor stimulation stabilizes NIK, which activates IKKα and induces phosphorylation and partial proteolysis of the NF-κB2 precursor p100 to p52. The resulting p52/RelB heterodimer translocates to the nucleus and regulates a distinct transcriptional program. Functionally, this pathway controls lymphoid organ development, B-cell survival and maturation, adaptive immune responses, and osteoclast differentiation. Its persistent or excessive activation may therefore sustain chronic inflammation, autoimmunity, and bone resorption [85].

#### 2.9.2. Role of NF-κB in Periodontitis

NF-κB is a key regulator of periodontal inflammation and tissue destruction. It controls the expression of numerous cytokines, chemokines, adhesion molecules, and enzymes involved in inflammatory responses, including IL-1β, IL-6, TNF-α, and other mediators implicated in periodontal disease progression. Increased NF-κB activity has been observed in periodontal lesions and is strongly associated with the severity of inflammatory tissue damage [15,86,87].

A complementary mechanism linking B-cell activity with NF-κB signaling in periodontitis involves the BAFF/BAFF-R axis. BAFF-R activates predominantly non-canonical NF-κB signaling, supporting B-cell survival and maturation [35]. BAFF expression is increased in periodontal inflammation, and human gingival fibroblasts can serve as a local BAFF source [88]. In experimental periodontitis, BAFF blockade reduced TNF-α expression, osteoclast formation, and alveolar bone loss [89]. These findings identify BAFF/BAFF-R/NF-κB signaling as a potential host-modulation target; however, current evidence remains mainly preclinical and does not yet support routine clinical use in periodontitis.

Clinical studies have demonstrated elevated expression of NF-κB subunits p50 and p65, accompanied by reduced levels of the inhibitory protein IκB, in periodontal tissues from patients with periodontitis compared with healthy controls [86]. Moreover, activation rates of NF-κB have been reported to be substantially higher in diseased periodontal tissues, supporting its pivotal role in disease pathogenesis. Experimental inhibition of NF-κB signaling reduces the production of pro-inflammatory cytokines, including IL-1β, IL-6, and TNF-α, thereby attenuating periodontal inflammation [87].

The importance of NF-κB signaling has also been demonstrated in human periodontal ligament stem cells (hPDLSCs), where inhibition of NF-κB activation significantly decreases the expression of IL-6 and IL-8 [90]. Conversely, activation of NF-κB through visfatin-mediated phosphorylation of p65 enhances the production of pro-inflammatory cytokines, further amplifying inflammatory responses within periodontal tissues [91].

Non-coding RNAs can modulate NF-κB-related inflammatory and osteoclastogenic signaling during periodontitis. The lncRNA Nron promotes nuclear transport of NF-κB repressing factor (NKRF), thereby suppressing osteoclastogenesis and alveolar bone resorption, whereas miR-21 can suppress NF-κB activation; reduced miR-21 expression is associated with lower IκB and enhanced inflammatory signaling [92,93].

NF-κB is also integral to bone metabolism. Combined deficiency of NF-κB1 and NF-κB2 impairs osteoclast and B-cell development and produces severe skeletal abnormalities, whereas experimental suppression of NF-κB has increased bone formation in ovariectomized mice [94,95].

A crucial mechanism underlying periodontal bone loss involves receptor activator of nuclear factor κB ligand (RANKL)-mediated osteoclastogenesis. Elevated RANKL expression in periodontitis promotes osteoclast activity and alveolar bone resorption, whereas osteoprotegerin acts as a decoy receptor that limits RANK activation on osteoclast precursors [16,96].

The non-canonical NF-κB pathway contributes to chronic periodontitis by linking adaptive immune responses with bone metabolism. RANKL-RANK signaling activates both canonical and non-canonical NF-κB pathways. In osteoclast precursors, the NIK-IKKα-NF-κB2 axis promotes p100 processing, p52/RelB activation, NFATc1 induction, osteoclast differentiation, and alveolar bone resorption. In experimental periodontitis, loss of NIK function or local administration of NIK inhibitors, including Cpd33 and NIK-SMI1, reduced non-canonical NF-κB activation, osteoclast formation, inflammatory mediator expression, and alveolar bone loss [97,98]. Thus, the NIK-IKKα-NF-κB2 axis may represent a more selective therapeutic target than global NF-κB blockade; however, the available evidence remains preclinical and requires confirmation in human studies.

Pirfenidone suppressed NF-κB activation, inflammatory cytokine production, and alveolar bone loss in a murine ligature-induced periodontitis model [99]. TCF7L2-associated NF-κB signaling has also been implicated in cementum generation [100]. These findings remain preclinical and do not support routine periodontal use.

Collectively, these findings highlight the central role of NF-κB in coordinating inflammatory responses, immune regulation, and bone remodeling during periodontitis. Consequently, therapeutic strategies targeting NF-κB signaling may represent promising approaches for limiting periodontal tissue destruction and promoting periodontal regeneration [15,97,98,99,100].

## 3. Conclusions

The JAK/STAT and NF-κB pathways integrate cytokine signals that sustain inflammation, osteoclastogenesis, and periodontal tissue destruction. Periodontitis provides a useful translational model because microbial dysbiosis, citrullination, and genetically conditioned immune responses may connect local mucosal disease with systemic autoimmunity, particularly ACPA-positive RA. Approved cytokine- and JAK-targeted therapies confirm the clinical value of pathway modulation, whereas plant-derived compounds remain exploratory and require better-defined mechanisms, formulations, dosing, and safety. Future studies should test selective and preferably local adjunctive strategies without compromising protective immune functions.

## Figures and Tables

**Table 1:** Selected conventional inhibitors and modulators of inflammatory signaling discussed in this review.

Principal Target/Class	Representative Inhibitor(s)	Mechanism of Action	Main Clinical Applications	Major Limitations	Refs.
TNF-α	Adalimumab; certolizumab pegol; etanercept; golimumab; infliximab	Neutralization of soluble or membrane TNF-α, or blockade of receptor engagement	RA; psoriasis; PsA; inflammatory bowel disease	Serious infections; tuberculosis reactivation; demyelinating disease; caution in heart failure	[31,32,33,34]
B cells/BAFF	Rituximab; belimumab	CD20-positive B-cell depletion or neutralization of soluble BAFF	Refractory RA; SLE; lupus nephritis	Infusion reactions; infections; HBV reactivation; rare PML	[22,35,36,37]
IL-6R	Tocilizumab; sarilumab	Blockade of membrane-bound and soluble IL-6R	RA and other inflammatory disorders	Infections; neutropenia; hepatic and lipid abnormalities; gastrointestinal perforation	[9,23,38]
IL-1/IL-1R	Anakinra; canakinumab; rilonacept	IL-1 receptor antagonism or neutralization of IL-1β	Autoinflammatory disorders; Still’s disease; RA	Infections; neutropenia; injection-site reactions	[2,10]
IL-17A	Secukinumab; ixekizumab	Neutralization of IL-17A	Psoriasis; PsA; axial spondyloarthritis	Mucocutaneous candidiasis; infections; possible exacerbation of inflammatory bowel disease	[24,39]
IL-12/IL-23 p40	Ustekinumab	Neutralization of the shared p40 subunit	Psoriasis; PsA; inflammatory bowel disease	Infections; injection-site reactions	[25]
CD80/CD86–CD28 co-stimulation	Abatacept	CTLA-4–Ig-mediated blockade of T-cell co-stimulation	RA; juvenile idiopathic arthritis; PsA	Infections; administration-related reactions	[40]
JAK1/JAK2	Baricitinib; ruxolitinib	ATP-competitive inhibition of JAK1/2-dependent STAT activation	RA or atopic dermatitis; myeloproliferative neoplasms (drug-dependent)	Infections; herpes zoster; hematologic and lipid abnormalities; MACE or VTE risk in susceptible patients	[29,30,51,52]
JAK1/JAK3	Tofacitinib	Inhibition of cytokine signaling mediated mainly by JAK1 and JAK3	RA; PsA; ulcerative colitis	Serious infections; herpes zoster; malignancy; MACE; VTE	[29,30,53]
Preferential JAK1	Filgotinib; upadacitinib; abrocitinib	Preferential blockade of JAK1-dependent cytokine signaling	RA; atopic dermatitis; inflammatory bowel disease (drug-dependent)	Infections; hematologic, hepatic, and lipid abnormalities; cardiovascular risk depends on patient profile	[29,30,54,55,56]
JAK2 and related kinases	Fedratinib; pacritinib; gandotinib	Preferential JAK2 inhibition; additional kinase effects vary by agent	Myelofibrosis and other myeloproliferative neoplasms; some agents remain investigational	Myelosuppression; thrombocytopenia; anemia; gastrointestinal and neurological toxicity	[57,58,59,60]
Other JAK inhibitors	Decernotinib; peficitinib; momelotinib; gusacitinib; delgocitinib	Selective or multikinase JAK inhibition; some agents also target SYK or ACVR1	Rheumatic, hematologic, and dermatologic disorders; some uses remain investigational	Heterogeneous safety profiles; infections; hematologic abnormalities	[61,62,63,64,65,66,67]
Direct STAT strategies	Peptide-based agents; decoy ODNs; ASOs; siRNA	Interference with phosphorylation, dimerization, DNA binding, or expression	Predominantly preclinical or early clinical development	Poor stability; limited cellular permeability; delivery challenges	[27,28]

**Table 2:** Selected plant-derived compounds modulating the JAK/STAT signaling pathway.

Compound	Natural Source	Principal Molecular Target(s)
Artemisinin/Artesunate	Artemisia annua	STAT3 [77,84]
Catechins (EGCG)	Green tea (Camellia sinensis)	STAT3; Nrf2; NF-κB [78,82]
Celastrol	Tripterygium wilfordii	STAT3; NF-κB [79,83]
Curcumin	Curcuma longa	STAT3; NF-κB [68,80]
Resveratrol	Grapes, blueberries, peanuts	STAT3; NF-κB [69,81]

**Table 3:** Selected plant-derived compounds investigated in autoimmune disease.

Compound	Disease Context	Representative Finding	Evidence Level
Curcumin	Rheumatoid arthritis	Small randomized pilot study; improvement in disease-activity measures was reported [80].	Clinical, preliminary
Resveratrol	Rheumatoid arthritis	Adjunctive clinical study; reductions in disease activity and inflammatory markers were reported [81].	Clinical, preliminary
Catechins (EGCG)	Autoimmune arthritis	Reduced clinical and histological arthritis in mice, with IDO-positive dendritic cells, Treg expansion, and Nrf2 activation [82].	Preclinical
Celastrol	Rheumatoid arthritis model	Suppressed inflammation and joint damage in rat adjuvant-induced arthritis [83].	Preclinical
Artesunate	SLE/lupus nephritis	Improved serological and renal outcomes and reduced BAFF-related activity in MRL/lpr mice [84].	Preclinical

## Data Availability

No new data were created or analyzed in this study. Data sharing is not applicable to this article.
